# Vasitis from Laparoscopic Inguinal Hernia Repair

**DOI:** 10.5334/jbsr.1523

**Published:** 2018-03-26

**Authors:** Willem Guillermo Calderon Miranda, Luis Rafael Moscote-Salazar, Paul M. Parizel

**Affiliations:** 1 Department of Radiology, Hospital General Doctor Manuel Gea González, National Autonomous University of Mexico, MX; 2 University of Cartagena, CO; 3 Department of Radiology, Antwerp University Hospital, University of Antwerp, BE

**Keywords:** Vasitis, TAPP, Tomography, Ultrasound

A 78-year-old man with a history of type 2 diabetes mellitus and arterial hypertension presented to the emergency department with right groin pain and fever. Just two days before, he had undergone laparoscopic transperitoneal inguinal hernia repair (TAPP) of a right-sided indirect inguinal hernia with fixation of mesh. Physical examination revealed swelling and painful palpation of the right groin. The patient had a total white blood cell (WBC) count 22,100 per microliter, neutrophilia and hemoglobin level of 16.7 g per deciliter. Inguinal ultrasonography demonstrated enlargement of the right spermatic cord with inflammation of the fat (Figure 1, arrows). A non-contrast computed tomography (CT) of the pelvis (Figure 2 A, B) revealed right-sided thickening of the spermatic cord and edema of the inguinal canal (blue arrows), both indicative of vasitis. Postoperative subcutaneous emphysema was noted (red arrows). The patient was treated non-invasively with broad spectrum antibiotics and analgesics. After two days, he was discharged with pain relief and without fever. As in the literature there is no report of infectious vasitis as a complication of TAPP, and we assume this is the first.

**Figure 1:**
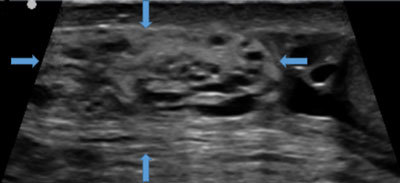
Sagittal grayscale US image shows a marked increase in the size and echogenicity of the right spermatic cord (arrows).

**Figure 2:**
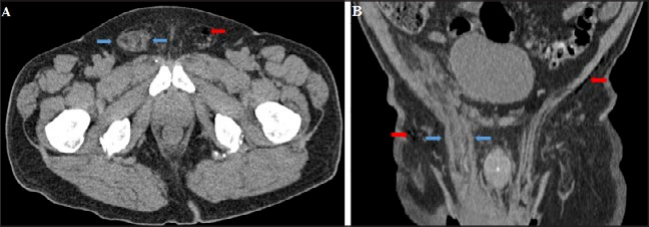
Unenhanced CT-scan of the pelvis, axial source image **(A)** and coronal reformatted image **(B)** shows the inflamed right spermatic cord, which cause distension of the inguinal canal (arrows blue). Subcutaneous emphysema and gas bubles within the left inguinal canal also were present (arrows red).

## Comment

Vasitis is an uncommon condition that can be misdiagnosed as incarcerated inguinal hernia due to a similar appearance at ultrasound, which leads to unnecessary surgeries. CT helps in the differentiation of vasitis from inguinal hernia because of the latter is clearly identifiable in multiplanar reconstructions [1].
